# Advance in the use of artificial intelligence of pulmonary nodule: evolution, trends, and future directions

**DOI:** 10.1097/JS9.0000000000003059

**Published:** 2025-07-15

**Authors:** Xue Li, Wenzheng Zhang, Jiliang Fang, Chunzhi Li, Hongsheng Lin, Yun Xu, Yufei Yang, Xueqian Wang

**Affiliations:** a Department of Oncology, Xiyuan Hospital, China Academy of Chinese Medical Sciences, Beijing, China; b Department of Oncology, Guang’anmen Hospital, China Academy of Chinese Medical Sciences, Beijing, China; c Graduate School, Beijing University of Chinese Medicine, Beijing, China; d Department of Radiology, Guang’anmen Hospital, China Academy of Chinese Medical Sciences, Beijing, China; e Department of Radiology, Xiyuan Hospital, China Academy of Chinese Medical Sciences, Beijing, China

**Keywords:** artificial intelligence, early-stage lung cancer, hot spots, pulmonary nodule, research trends

## Abstract

**Background:**

Timely detection and intervention for pulmonary nodules play a vital role in decreasing lung cancer-related deaths. Nevertheless, the precise differentiation between benign and malignant nodules continues to face a major clinical challenge. With the rapid progress of artificial intelligence (AI), significant improvements have been made in the detection, classification, and clinical decision-making related to pulmonary nodules. Although scholarly interest in this domain has surged in recent years, there is still a lack of comprehensive bibliometric studies that systematically map its current landscape and evolution. This study seeks to explore emerging research trends, highlight thematic focus areas, and analyze patterns of collaboration within the field of AI-assisted pulmonary nodule research over the past 20 years.

**Methods:**

A literature search was conducted in the Web of Science Core Collection to collect relevant studies published from 2005 to 2024 concerning the application of AI in pulmonary nodules. Bibliometric analysis was carried out using tools such as CiteSpace, VOSviewer, and the Online Analysis Platform of Literature Metrology to examine contributions from countries, institutions, authors, journals, keywords, and references.

**Results:**

A total of 1657 relevant publications were retrieved, reflecting a consistent upward trend in research output over the past two decades, with a marked acceleration observed after 2014. The leading contributors in terms of publication volume were China, the United States, and India. Shanghai Jiao Tong University stood out as the most prolific research institution. Analysis of keyword co-occurrence revealed several prominent thematic clusters, notably centered around Deep Convolutional Neural Network models, major diameter, lung nodule detection, false-positive reduction, cancer diagnosis, quantitative-semantic models, double reading, and clinical utility studies.

**Conclusions:**

This bibliometric study offers a thorough assessment of the scholarly landscape concerning AI applications in pulmonary nodule research, underscoring major developments and key contributors. The insights gained may serve as a strategic reference for researchers in the medical and AI fields, facilitating informed future directions. Notably, the intersection of AI and pulmonary nodule research is concentrated in the following areas: (1) Application of AI in pulmonary nodule detection and classification; (2) AI in malignancy risk prediction and growth modeling; (3) AI-driven development of drug efficacy evaluation metrics may be a future direction for pulmonary nodule treatment research.

## Introduction

Lung cancer has been the most common cancer for several decades, where there were about 2.2 million new cases and 1.8 million new deaths in 2020^[1]^. The survival rate of lung cancer decreased with the increase of stage, the 5-year survival rate of stage I lung cancer was 92%, but that of stage IV was only 38%^[2]^. Early-stage lung cancer screening is of great significance to reduce lung cancer mortality; the US National Lung Screening Clinical Trials showed a 20% reduction in lung cancer mortality with lung cancer screening^[3]^. Pulmonary nodule, as one of the common lesions in the lung, refers to small (<3 cm), focal, distinct radiographic densities surrounded by lung tissue^[4],^ which is one of the most important imaging findings of early-stage lung cancer. Studies have shown that the probability of malignancy increased with the diameter of the pulmonary nodule^[5]^. If the diameter of the pulmonary nodule was between 10 and 20 mm, the probability of malignancy was 33%–64%; while if the diameter of the pulmonary nodule was greater than 20 mm, the probability of malignancy was from 64% to 82%^[5]^. Nowadays, surgery and computerized tomography (CT)-based follow-up are the main intervention methods of pulmonary nodule^[6]^. Although some typical benign or malignant pulmonary nodules could be judged by manual reading, a considerable proportion of lesions could not be detected, diagnosed, and identified. Besides, due to the doctors’ visual fatigue, personal experience, and the level of care difference caused by economic differences, there is still quite a variation in the current clinical judgment of benign and malignant pulmonary nodules. Luckily, current studies have shown that AI could solve these problems and even outperform human judgment in some cases, which could assist clinicians in making final decisions^[7]^.

Surgery is the main treatment for early-stage lung cancer and high-risk pulmonary nodule. However, surgery is an invasive treatment, which would not only lead to fatigue, shortness of breath, pain, and other symptoms^[8,9]^, but also potentially increase the risk of complications to some extent, such as decreased lung function, cardiovascular complications, pulmonary embolism, acute lung injury, and respiratory failure^[10,11]^. This is also one of the main reasons why many patients with pulmonary nodules hesitate to undergo surgery. If benign pulmonary nodule is misdiagnosed as early-stage lung cancer and undergoes surgery, this situation might unnecessarily increase the patient’s symptom burden and reduce their quality of life. While if lung cancer is misjudged as benign pulmonary nodules, long-term follow-up or neglect might cause lesion progression, delay the optimal treatment period, and reduce survival^[12]^. These common clinical situations justify the need to improve the detection rate of pulmonary nodules and the accurate diagnosis rate of lung cancer, to help patients make correct clinical decision-making; AI may be a promising auxiliary tool^[13]^.

AI is a system that mimics human intelligence, which is characterized by the ability to perceive, reason, find meaning, generalize, learn from experience, solve problems, and make decisions^[14]^. CT imaging is one of the most important detection methods for pulmonary nodules. In recent years, the application of AI in pulmonary nodules has excelled in many aspects, such as identification, diagnosis, surgical planning, and follow-up^[15–18]^. AI is centered on developing algorithms to perform complex tasks in the clinical setting of radiology, to aid in the interpretation of image data quickly and effectively. In addition, AI could overcome the limitations of conventional imaging that relies on visual judgment, which could transform images into massive data features that could be mined, to characterize the heterogeneity and micro-environment objectively and quantitatively within tumors. Studies have shown that the capabilities of AI were comparable to experts or in some cases exceeded the performance of experts^[19];^ for example, AI could help clinicians reduce the misdiagnosis rate of pulmonary nodules effectively^[20]^. Moreover, AI could improve the accuracy of clinical decision-making by learning from massive data. The research field of AI in pulmonary nodules includes the combination of medicine and engineering, which would not only provide help for the development of interdisciplinary research and the advances in medicine, but also help clinicians to make more objective and accurate medical decisions^[21]^. Currently, numerous AI software tools have been applied to assist in clinical decision-making for pulmonary nodules and have achieved promising results. These tools typically integrate advanced AI algorithms to support detection, diagnosis, treatment planning, and monitor, such as Lung-RADS Assistant, LUNA16 Dataset Tools, and Arterys Lung AI^[22–24]^. However, the Fleischner guideline indicated that current AI-assisted CT still has the disadvantage of showing a high false-positive rate in the detection of pulmonary nodules^[25]^. Additionally, the integration of multimodal data (e.g. genomics and pathology) with AI still faces challenges, including limitations in existing methodologies (e.g. handling high-dimensional yet sparse genomic data) and a lack of interpretability^[26]^. The black box problem of deep learning algorithms has also become one of the challenges for AI^[27]^. In this context, although AI has shown great advantages and potential in the field of pulmonary nodules, there are still many problems at the intersection of medicine and engineering to be further solved as this field evolves.

**HIGHLIGHTS**

- First bibliometric analysis focused on artificial intelligence applications in pulmonary nodules.
- Reveals key trends and emerging hotspots using advanced bibliometric methods.
- Publications have grown steadily from 2005 to 2024, accelerating after 2014.
- Keyword analysis highlights core themes such as deep learning models, nodule size, detection accuracy, cancer diagnosis, and clinical relevance.

Bibliometrics, based on mathematics and statistics, is one way to analyze massive heterogeneous literature. So far, despite the increasing number of publications on AI in pulmonary nodules, there is a lack of a systematic and comprehensive bibliometric analysis that not only outlines the evolution of research trends but also identifies the specific challenges, knowledge gaps, and potential solutions in this interdisciplinary field. In this study, we collected relevant publications on AI in pulmonary nodules from the Web of Science Core Collection (WoSCC) database, and determined the countries, institutions, authors, and journals, to provide information for researchers to select cooperation partners. Besides, this study aims to address the following key issues: (1) What are the core technological and clinical challenges that remain unresolved in AI-assisted pulmonary nodule research? (2) Which thematic areas are emerging as frontiers and have the potential to drive breakthroughs? (3) How can the mapping of global research efforts provide strategic guidance for clinicians, engineers, and decision-makers? By answering these questions, this study seeks to offer not only an overview of the current research landscape but also practical guidance to facilitate collaboration between medicine and artificial intelligence, promoting more efficient clinical translation and future development. The work has been reported in line with the TITAN criteria^[28]^.

## Methods

### Data sources

The WoSCC was selected as the primary data source for this bibliometric analysis for the following reasons. First, WoSCC is widely regarded as one of the most authoritative, comprehensive, and internationally recognized academic databases. Second, it offers detailed bibliographic records that are fully compatible, which facilitates the subsequent analysis of knowledge maps. Third, it is considered that AI in pulmonary nodule research involves a multidisciplinary field^[29,30]^. Therefore, WoSCC, which encompasses both SCIE and SSCI, was determined to be the most suitable and reliable source for data retrieval in the present study. Fourth, the selection of WoSCC is consistent with methodological precedents established in previous bibliometric studies, particularly those conducted by Chaomei Chen, the developer of CiteSpace^[31–33]^.

### Search strategies

Medical Subject Headings (MeSH) are widely used as a controlled vocabulary for indexing and searching biomedical literature in PubMed, and to ensure a comprehensive search, we employed MeSH^[34]^. Additionally, Entry Terms, which refer to synonymous or closely related terms associated with MeSH terms^[35]^, were also utilized to construct search formulas. The final search strategy used in WoSCC was as follows: TS = (artificial intelligence OR AI OR deep learning OR machine learning OR computational intelligence OR Convolutional Neural Network OR CNN OR radiomics) AND TS = (lung nodule OR pulmonary nodule OR chest nodule). Besides, no language filter was applied during the initial search, but only English-language articles were retained during screening.

**Figure 1.**
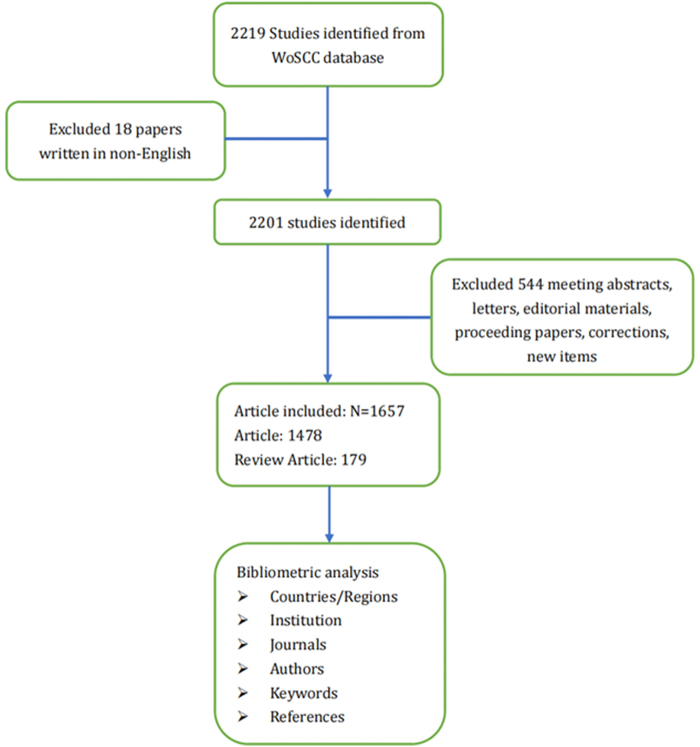
Frame flowchart for the detailed selection criteria and bibliometric analysis step in the WoSCC database.

As illustrated in Figure 1, articles were retrieved using the topic search (Topics, TS) function in WoSCC. This study encompasses the majority of publications on AI for pulmonary nodule available in this database to date, including English articles published from January 1, 2005 to September 15, 2024. After the initial search, a total of 2201 publications were retrieved.

### Data extraction, cleansing, and standardization

For bibliometric analysis, records were exported as Plain Text Files, including information on authors, titles, sources, citations, and abstracts. The process of data selection strictly adhered to the PRISMA guidelines^[36]^. Prior to analysis, data cleaning was conducted to improve consistency and reduce bias. Variants of author names and institutional names (e.g. “Harvard Univ” vs “Harvard University”) were manually standardized. Countries with different naming conventions (e.g. “USA” vs “United States”) were unified. Duplicate records (based on title and DOI) were removed using EndNote and manually verified. To ensure methodological reliability and minimize subjective bias, two researchers independently performed the literature screening, keyword clustering, and thematic analysis. Any disagreements were resolved through discussion or by consulting a third reviewer. Additionally, a random sample of 50 records was manually reviewed to verify the accuracy of automated cluster classification and citation mapping.

Document types are articles and review articles. Eligible publications met these criteria: (1) Published between January 1, 2005 and September 15, 2024; (2) written in English; (3) classified as articles or reviews. Exclusions included meeting abstracts, letters, editorials, proceedings, corrections, news items, and unpublished documents lacking sufficient detail. After the initial search, a total of 1657 publications were retrieved.

### Bibliometric tools and visualization analysis

After screening all eligible publications, data were analyzed and visualized using CiteSpace, VOSviewer, and the Online Analysis Platform of Literature Metrology. The characteristics of all identified papers were thoroughly recorded. The impact factor (IF), a key metric for assessing academic influence, was obtained from the *Journal Citation Reports* (JCR). The H-index, sourced from the WoSCC, reflects the number of publications with at least h citations. The Online Analysis Platform of Literature Metrology was used to analyze publication volumes and growth trends. CiteSpace (version 6.1.R6) facilitated citation analysis, co-citation analysis, co-authorship networks, citation bursts, journal dual-map overlays, and keyword timeline clusters, with most results visualized as interconnected nodes and links. VOSviewer (version 1.6.18) was primarily employed for reference cluster analysis. Different nodes represent different elements, and the differences in clustering are indicated by different colors in the maps.

### Parameter setting

CiteSpace settings were configured as follows: (1) Time slicing: 2005–2024, 1-year per slice; (2) Node types: Country, Institution, Author, Journal, Keyword, Reference; (3) Pruning: Pathfinder or Minimum Spanning Tree; (4) Clustering algorithm: Log-likelihood ratio. In VOSviewer, a minimum occurrence threshold of five was applied for keyword co-occurrence mapping, and the layout was optimized using the LinLog/modularity-based clustering algorithm.

### Ethics

Given that the data originated from the publicly accessible WoSCC database, obtaining ethical approval from an institutional review board was deemed unnecessary.

## Results

### Publishing trend analysis

**Figure 2.**
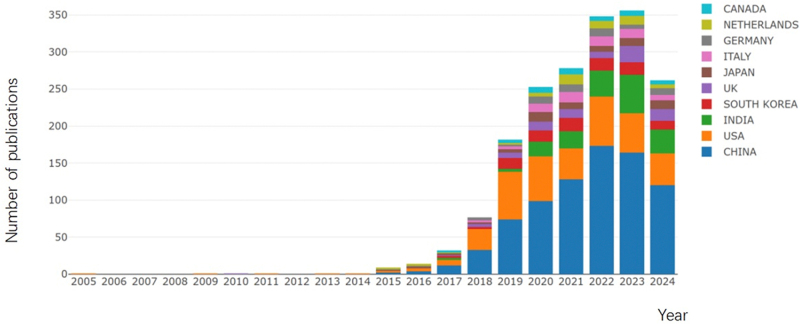
Distribution of annual year and growth trends of the top 10 countries/regions.

A total of 1657 publications were identified. These comprised 1478 original articles (89.2%) and 179 reviews (10.8%) (Fig. 1). Collectively, these articles received 34 344 citations, with an average of 21 citations per article, and an H-index of 81. As shown in Figure 2, research on AI-based pulmonary nodules has grown significantly over the past two decades. Before 2014, annual publication numbers increased gradually, with no notable trends. However, from 2015 to 2021, the field experienced a rapid surge in publications. Besides, Figure 2 also illustrates publication trends among the top 10 contributing countries/regions. China ranked first with a rapid growth trajectory, followed by the United States, which maintained steady output. India also showed a noticeable increase in publication numbers.

### Publications and collaborative networks of countries/regions, institutions, authors, and journals

**Figure 3.**
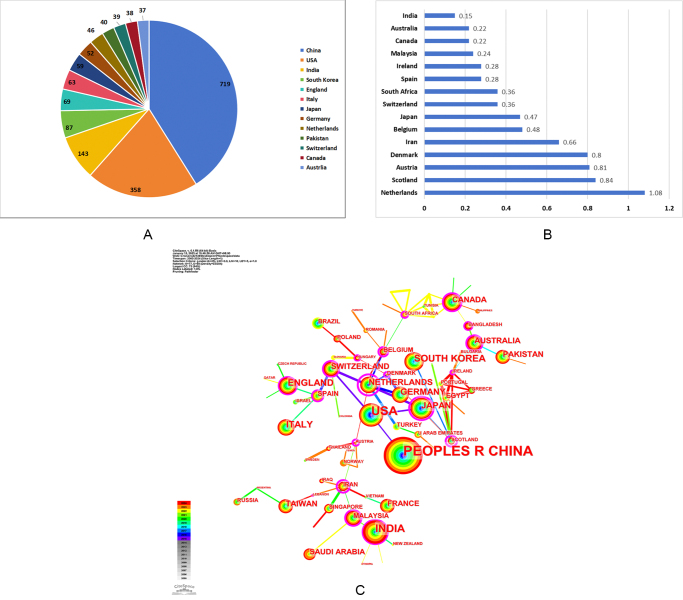
Each country/region’s contribution to the research of AI-based pulmonary nodule. (A) Number of publications by country. (B) Intermediary centrality of countries. (C) collaborative networks of countries.

**Figure 4.**
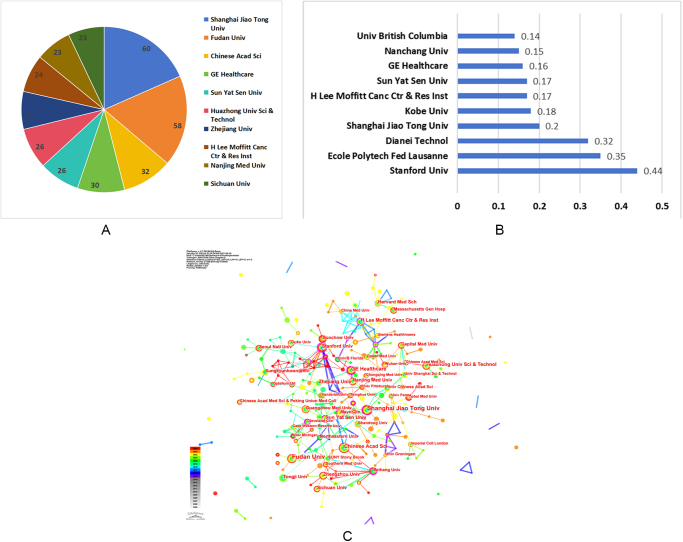
Each institution’s contribution to the research of AI-based pulmonary nodule. (A) Number of publications by institution. (B) Intermediary centrality of institutions. (C) collaborative networks of institutions.

**Figure 5.**
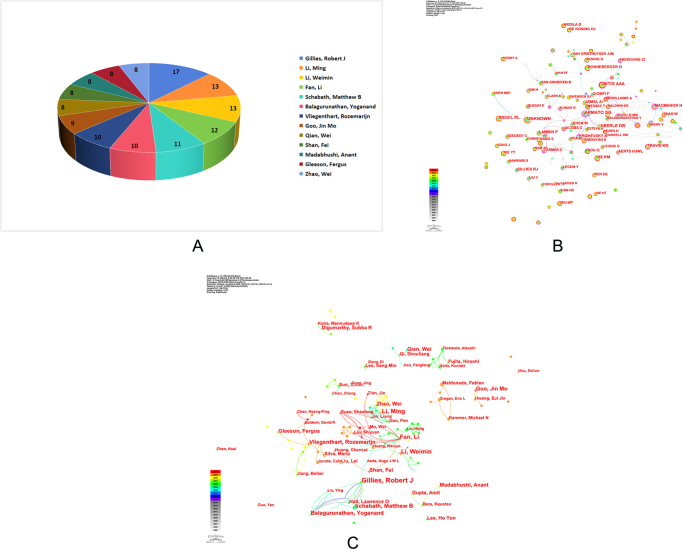
Each author’s contribution to the research of AI-based pulmonary nodule. (A) Number of publications by author. (B) Collaborative networks of authors. (C) Collaborative networks of co-cited authors.

**Figure 6.**
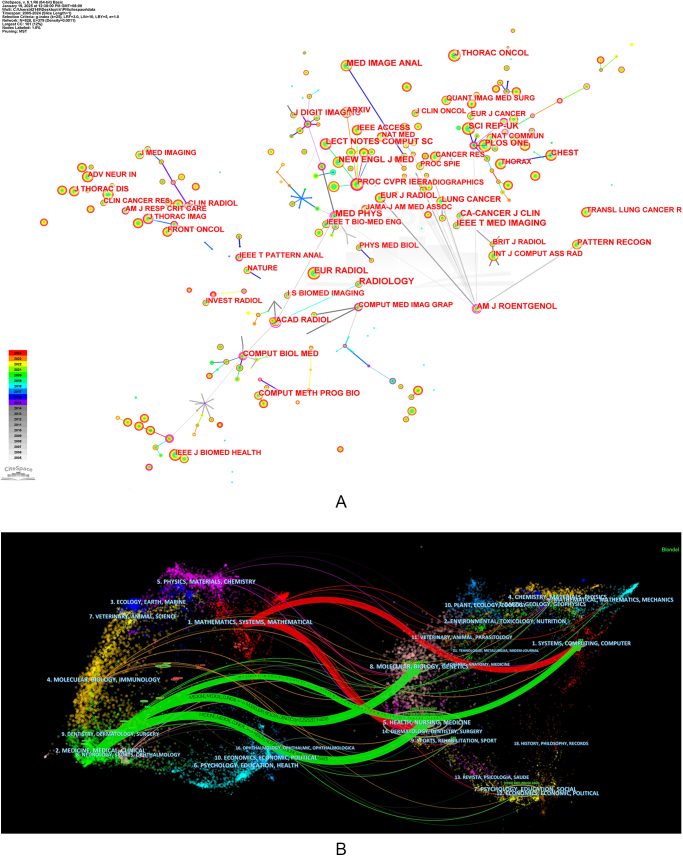
Each journal’s contribution to the research of AI-based pulmonary nodule. (A) Collaborative networks of journal. (B) A journal dual-map overlap. Citing journals are on the left, cited journals are on the right. Colored lines represent citation associations.

We examined the quantity of publications and collaborative networks of countries, regions, institutions, and authors involved in AI and pulmonary nodule research. The larger the node in the graph, the greater the number of publications. The purple outer circle denoted a centrality value higher than 0.1 for the intermediary. Moreover, a larger centrality value indicated increased collaboration between the node and other nodes. Detailed information could be referred to Figures 3–6.

## Analysis of national publications and collaborations

The study analyzed the publication count (Fig. 3A), mediator centrality (Fig. 3B), and collaborative networks (Fig. 3C) of research on AI-based pulmonary nodules in various countries/regions. As shown in Figure 3A, China led with the highest number of publications (719), followed by the USA (358) and India (143). The rest of the countries/regions had less than 100 publications. Besides, as shown in Figure 3C, in terms of centrality – an indicator of influence in the collaborative network – the Netherlands (1.08), Scotland (0.84), and Austria (0.81) ranked highest.

Countries such as the Netherlands, Scotland, and Austria, despite not having the highest number of publications, demonstrated strong intermediary roles within the international collaboration network. For instance, the Netherlands served as a major bridge, connecting various research hubs worldwide. Similarly, Scotland and Austria also acted as vital nodes linking multiple institutions across continents, suggesting that these countries facilitate knowledge exchange and multinational collaborations. In contrast, China, although leading in quantity, showed relatively lower centrality values, indicating more intra-national rather than inter-national collaborations. Specific country information about publication quantity could be found in Supplemental Digital Content 1 Table S1, available at: http://links.lww.com/JS9/E678.

## Analysis of institutional publications and collaborations

Figure 4A displays publications from institutions, while Figure 4B demonstrates the significance of institutions acting as intermediaries. Figure 4C illustrates a network diagram depicting collaboration between institutions. As shown in Figure 4A, Shanghai Jiao Tong University led with the highest number of publications (60), followed by Fudan University (58), and the Chinese Academy of Sciences (32). Stanford University (0.44), Ecole Polytechnique Fédérale de Lausanne (0.35), and Dianei Technol (0.32) exhibited the highest centrality (Fig. 4B). The institutional collaboration network (Fig. 4C) revealed a relatively dense structure, with several institutional clusters primarily formed around institutions with high centrality. Notably, Shanghai Jiao Tong University and Fudan University, which led in publication counts, also formed dense collaborative clusters with nearby or affiliated research centers. However, high-centrality institutions such as Stanford University and EPFL (École Polytechnique Fédérale de Lausanne) played a key intermediary role by bridging geographically distant research groups, suggesting that they contribute to more globalized knowledge diffusion in this field. Specific information regarding the quantity of published works and the significance of institutions could be found in Supplemental Digital Content 1 Table S2, available at: http://links.lww.com/JS9/E678.

## Analysis of authors and co-cited authors

Publications from authors and the collaboration network between the authors were shown in Figure 5A. Figure 5B illustrates the collaborative network of authors, while Figure 5C illustrates the collaborative networks of co-cited author. As shown in Figure 5A, the most prolific contributors were Gillies, R.J. (17 publications), Li, M. (13 publications), and Li, W.M. (13 publications). In the co-citation analysis (Fig. 5B), the most frequently co-cited authors were Armato, S.G. (450 citations), Aberle, D.R. (367 citations), Setio, A.A.A. (339 citations), and Macmahon, H. (223 citations). Notably, Gillies, R.J., a professor at Moffitt Cancer Center, appeared in the list of co-authorship and co-citation authors, underscoring his significant influence in this area of study. While there was collaboration among multiple authors with a significant number of publications, the level of cohesion of the collaboration may not be high (Fig. 5B and C). Figure 5B shows that although some author clusters were formed (e.g. Gillies, R.J. and collaborators), overall author collaboration appeared relatively fragmented, with several small groups working in isolation. Figure 5C further highlights key co-cited authors such as Armato, S.G. and Setio, A.A.A., who, while not always the most prolific, were frequently referenced together, indicating their foundational role in establishing methodological frameworks in AI-based pulmonary nodule detection. The sparse connections between groups imply a need for enhanced international and interdisciplinary author collaboration to advance the field more cohesively. Table 1 provides detailed information on these influential authors.

**Table 1:** Top 10 co-authorship and co-citation authors contributing to publications in the research of AI in pulmonary nodules

Rank	Author	Country	Count	Co-cited author	Country	Count
1	Gillies, R.J.	USA	17	Armato, S.G.	USA	450
2	Li, M.	China	13	Aberle, D.R.	USA	367
3	Li, W.M.	China	13	Setio, A.A.A.	Germany	339
4	Fan, Li.	China	12	Macmahon, H.	Ireland	223
5	Schabath, M.B.	USA	11	Lambin, P.	Netherlands	215
6	Vliegenthart, R.	Netherlands	10	He, K.M.	China	214
7	Balagurunathan, Y.	USA	10	Siegel, R.L.	USA	209
8	Goo, J.M.	South Korea	9	Gillies, R.J.	Australia	202
9	Qian, Wei.	China	8	Aerts, H.J.W.L.	USA	196
10	Shan, Fei.	China	8	Jemal, A.	USA	195

## Analysis of journal publications and collaborations

As shown in Figure 6A, journals such as *Radiology, European Radiology*, and *American Journal of Roentgenology* showed high co-citation frequencies and betweenness centrality, indicating their central position in the network. Computational journals *like IEEE Transactions on Medical Imaging, Medical Image Analysis*, and *Computers in Biology and Medicine* also rank prominently, underscoring the field’s interdisciplinary nature. Notable clusters include journals on thoracic oncology (*Lung Cancer, Chest, Thorax*) and broader scientific outlets (*PLOS One, Scientific Reports*), indicating both clinical focus and cross-disciplinary interest.

Figure 6B shows the dual-map overlay of journals related to AI in pulmonary nodule research. The left side represents citing journals, and the right side shows cited journals, with colored lines indicating citation paths across disciplines. Two main citation trajectories are evident. One path links journals in medicine and clinical research to those in molecular biology and genetics, suggesting that clinical studies draw on biological foundations. Another path connects computer science and mathematics journals with computational and biomedical fields, reflecting the methodological contribution of AI. The presence of multiple interconnecting paths underscores the multidisciplinary nature of AI applications in pulmonary nodule research, involving collaboration among computer science, bioinformatics, clinical medicine, and radiology. This cross-disciplinary citation structure indicates that advances in this field are driven by the integration of algorithmic innovation with biomedical application scenarios. Table 2 lists the top 10 co-citation journals ranked by the number of publications and their 2023 IF, most of which are Q1 journals according to the JCR.

**Table 2:** Top 10 co-citation journals contributing to publications in the research of AI-based pulmonary nodule

Ranking	Co-cited Journal	Counts	Centrality	IF (2023)#	JCR
1st	*Radiology*	1006	0	12.1	Q1
2nd	*Medical Physics*	781	1.06	3.2	Q1
3rd	*European Radiology*	729	0	4.7	Q1
4th	*Scientific Reports*	649	0.04	3.8	Q1
5th	*IEEE Transactions on Medical Imaging*	639	0	8.9	Q1
6th	*New England Journal of Medicine*	590	0	96.3	Q1
7th	*Medical Image Analysis*	586	0	10.7	Q1
8th	*CA-A Cancer Journal For Clinicians*	559	0.09	521.6	Q1
9th	*Plos One*	551	0.01	2.9	Q1
10th	*Lecture Notes in Artificial Intelligence*	508	0.11	–	–

### Analysis of keywords occurrence and cluster

**Figure 7.**
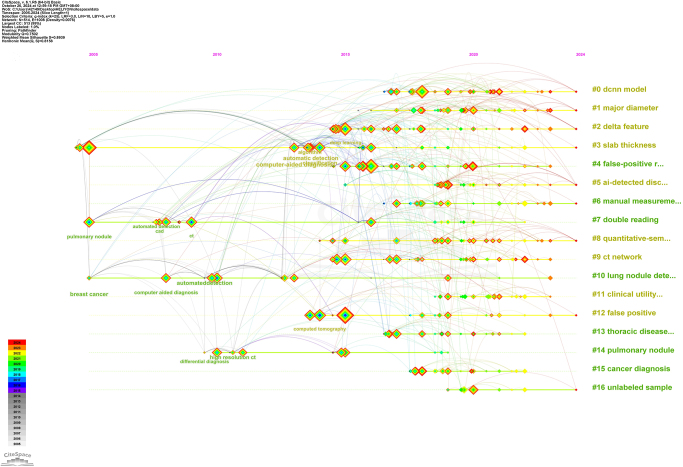
Timeline view of keyword clusters. Labels on the right represent the clusters, a node represents a keyword. Nodes with red tree rings refer to keywords with citation bursts.

Keywords encapsulated the core themes of research articles and provided insights into emerging trends and evolving priorities in the field. Table 3 presents the top 20 most frequent keywords and the keywords with the highest centrality. Besides, as shown in Figure 7, we conducted a cluster analysis, which provided a timeline visualization of keyword evolution, offering insights into how research priorities have shifted over time. The specific keyword labels included Deep Convolution Neural Network (DCNN) model, major diameter, delta feature, false-positive reduction, slab thickness, manual measurement, AI-detected discrepancies, double reading, CT network, quantitative-semantic model, clinical utility studies, lung nodule detection, thoracic diseases, false positive, cancer diagnosis, pulmonary nodule, and unlabeled sample. This timeline integrated temporal data with keyword clusters, making it possible to trace the field’s developmental trajectory. The size and position of nodes on the timeline indicated the prominence and initial appearance of keywords, while solid lines represented the duration of research focus on specific topics. These clusters reflected the primary research directions in the field. Among them, DCNN refers to deep convolutional neural networks, which have become a research hotspot in recent years for the automatic identification of pulmonary nodule. The quantitative-semantic model represents studies focused on the extraction of quantitative imaging features of pulmonary nodules. Major diameter and delta feature demonstrate the application of AI systems in assessing the malignancy potential of nodules using feature parameters. Moreover, the false positive rate remains a key area of ongoing concern. Detailed information about these clusters was presented in Supplemental Digital Content 1 Table S3, available at: http://links.lww.com/JS9/E678.

**Table 3:** Top 20 keywords contributing to publications in the research of AI-based pulmonary nodules

Ranking	Keywords	Count	Keywords	Centrality
1	lung cancer	425	computer-aided diagnosis	1.28
2	cancer	253	lung nodule	1.11
3	machine learning	114	computed tomography	0.83
4	model	76	segmentation	0.81
5	validation	75	clustered microcalcification	0.80
6	solitary pulmonary nodule	45	MR	0.80
7	feature extraction	44	chest	0.66
8	malignancy	38	pulmonary nodule	0.51
9	mortality	38	prediction	0.50
10	risk	37	soft computing	0.48
11	signature	36	automatic detection	0.47
12	network	34	detection system	0.44
13	artificial intelligence	32	evolution	0.38
14	ground glass nodule	30	disease	0.37
15	lung neoplasm	27	diagnosis	0.34
16	chest radiography	25	texture analysis	0.33
17	lesion	21	database consortium	0.31
18	benign	19	performance	0.31
19	tumor heterogeneity	18	tumor motion	0.28
20	feature selection	16	computer-aided detection	0.25

### Research hot spots and trend analysis

#### Analysis of highly co-cited references

**Figure 8.**
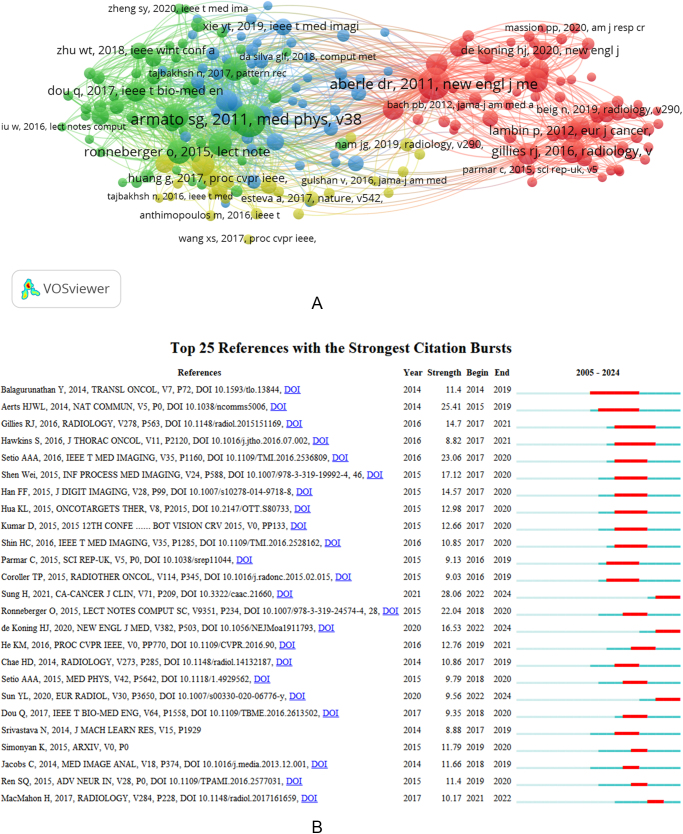
Each reference’s contribution to the research of AI-based pulmonary nodule. (A) Cluster mapping of highly co-cited literature. (B) Top 25 references with the strongest citation bursts. Red line refers to citation bursts detection, which indicated the start year, end year, and duration of the bursts.

We employed VOSviewer to plot the co-cited references, revealing that a total of 38 659 references had been cited. When setting the minimum citation time to 30, the resulting number of documents in the analysis was reduced to 206. Figure 8A displays the final relationship graph. The highly co-cited references in the network graph could be categorized into four clusters, each represented by a different color: red, green, blue, and yellow. The red cluster mainly focused on the development of AI algorithms and model architectures, including foundational work on convolutional neural networks (CNNs) and U-Net architectures. The green cluster centered on clinical trials and screening outcomes, particularly low-dose CT (LDCT) screening and early detection of lung cancer. The blue cluster highlighted radiomics and quantitative imaging biomarkers, which are critical for AI-based feature extraction and prognosis prediction. The yellow cluster included studies on false-positive reduction and detection sensitivity, emphasizing the clinical utility and limitations of automated systems. The highly co-cited references were included in Supplemental Digital Content 1 Table S4, available at: http://links.lww.com/JS9/E678.

#### Analysis of highly cited references

The top 10 most-cited references on AI-based pulmonary nodule research are listed in Supplemental Digital Content 1 Table S4, available at: http://links.lww.com/JS9/E678. All references collectively received 5694 citations, accounting for 16.2% of total citations. The most-cited reference was “End-to-end lung cancer screening with three-dimensional deep learning on low-dose chest computed tomography” by Tse D. *et al*, published in *Nature Medicine* (IF = 82.9) in 2019^[18]^. This study garnered 750 total citations, averaging 150 citations annually, which highlights its influence. Meanwhile, “UNet++: Redesigning Skip Connections to Exploit Multiscale Features in Image Segmentation” by Liang JM *et al*, published in *IEEE Transactions on Medical Imaging* in 2020, had the highest annual citation average (169.75), underscoring its significance^[37]^.

#### Analysis of cited literature bursts

Citation burst reflects a large change in the citation frequency of a cited document in each period. A red line indicates the citation burst detection. This line shows the start year, end year, and the duration of each burst, reflecting periods of significant impact in the field. Figure 8B presents the top 25 references with the strongest citation bursts. The highest burst value (28.06) was achieved by Sung H, 2021, *CA: A Cancer Journal for Clinicians*, which peaked between 2022 and 2024^[1]^. Notably, the citation bursts for three publications remain ongoing – “Radiomics for lung adenocarcinoma manifesting as pure ground-glass nodules: invasive prediction,” “Reduced Lung-Cancer Mortality with Volume CT Screening in a Randomized Trial” and “Global Cancer Statistics 2020: GLOBOCAN Estimates of Incidence and Mortality Worldwide for 36 Cancers in 185 Countries”^[1,38,39]^. These bursts suggest sustained interest in early diagnosis, screening effectiveness, and the global epidemiology of lung cancer, reinforcing the interdisciplinary relevance of AI in public health and clinical research.

## Discussion

The development of AI has led to rapid advancements in pulmonary nodule research, with numerous significant achievements in the past 20 years. However, the rapid increase in publications within this field presents a challenge for scientists aiming to identify and track emerging trends and research frontiers. Bibliometric analysis serves as a valuable tool to synthesize and summarize the current research landscape, aiding in the identification of key areas of focus and frontiers. In this study, we analyzed 1657 publications on AI-based pulmonary nodules from 2005 to 2024, retrieved from the WoSCC database. The analysis reveals steady growth in the field over the past two decades, with a notable surge in publications after 2014 (Fig. 2). This growth may be closely related to factors such as the increased focus on lung cancer screening, the development of AI, and the growing attention to interdisciplinary research. Previous bibliometric studies have systematically examined research on pulmonary nodules, initially identifying a close association between pulmonary nodules and AI^[40]^. However, no study has conducted an in-depth and systematically analysis of the emerging hotspots and trends in AI-related pulmonary nodule research. Moreover, with the growing interest in AI, the number of studies in this field has increased rapidly over the past years. Therefore, to the best of our knowledge, this is the first bibliometric analysis specifically focusing on AI in pulmonary nodules in recent years. Our findings highlight the trends and research hotspots within the field, which are further discussed in detail below.

### Analysis of countries/regions and institutions

China and the United States collectively account for over 50% of global publications on AI in pulmonary nodules, underscoring their significant contributions to the field (Fig. 3A). China leads in publication volume, while the United States ranks second. However, the United States outperforms China in terms of H-index and total citations. This phenomenon suggests that while China leads in research output, the United States excels in research quality and impact, revealing its position as a global leader in this field. As is known to us, developed countries, particularly the United States, benefit from advanced technology, well-funded research environments, skilled professionals, and strong international collaboration, all of which drive academic productivity. Notably, some developing countries, such as China, India, and Pakistan, have also demonstrated significant academic influence, as shown in Figure 3.

The advancement of AI technology depends heavily on access to large datasets for training. China has seen a significant increase in publications since 2018, whereas the growth in the number of publications from the United States has remained relatively steady (Fig. 2). This phenomenon is particularly intriguing and warrants further in-depth discussion. China’s large population serves as a significant data resource for AI development. National policy support bolsters China’s leadership in this research field, including initiatives like the “Three-Year Plan for AI”^[41]^. Similarly, India’s position as the third-largest contributor to AI-based pulmonary nodule research is driven by its large population and growing focus on AI. The achievements made by China and India demonstrate the crucial role of supportive policies in driving AI innovation. It is noteworthy that discussing the development trajectory of the United States also provides valuable insights into the application of AI in the field of pulmonary nodules. The United States has a longer history in AI development, dating back to John McCarthy’s introduction of the concept at the 1956 Dartmouth Conference, which initially sparked widespread research interest. However, the United States experienced setbacks during the 1970s and 1980s when funding cuts slowed progress, leading to a so-called “AI winter”^[42]^. This phase lasted until technological advancements reignited interest in 2006. This also demonstrates from another perspective that achieving continuous progress in AI-based pulmonary nodule research requires that the relentless efforts of researchers be combined with sustained government support. Besides, among the top 10 institutions in publication volume (Fig. 4A), seven are from China and two from the United States, further demonstrating the dominance of these two nations in this field.

Beyond publication volume and citation metrics, the divergent trajectories of China and the United States in AI-based pulmonary nodule research can also be interpreted through the lens of their underlying AI ecosystem maturity and data governance structures. While China benefits from a vast population and government-endorsed datasets, challenges in data standardization, hospital interconnectivity, and ethical review systems create barriers to large-scale, multi-centered AI validation. Conversely, the U.S. leverages its strong data governance frameworks and institutionalized open science practices, such as National Institutes of Health (NIH)-funded TCIA and NLST datasets, which facilitate reproducibility and international collaborations – critical elements in high-impact research^[43,44]^.

Furthermore, the “application-driven” model of China, focusing on rapid deployment of AI-assisted diagnostic tools, contrasts with the “innovation-driven” approach of the U.S., which prioritizes foundational algorithm development and interdisciplinary model robustness. This reflects not only policy direction but also the stage of ecosystem development, where the U.S. possesses a more mature innovation pipeline, from research to commercialization.

From a global network perspective, the Netherlands, Scotland, and Austria have played a central role in international scientific collaborative networks, acting as a bridging node between multiple countries. In contrast, China’s research efforts remain largely domestically concentrated, limiting international co-authorship visibility and cross-cultural model generalization. This suggests that future advances may depend not only on research capacity but also on how countries position themselves within global AI knowledge networks.

### Authors and co-cited authors

Identifying influential researchers provides valuable direction and guidance for the advancement of AI-based pulmonary nodule research. In this field, Gillies R.J. from the Moffitt Cancer Center has the highest number of publications in co-authorship analysis, while Armato S.G. from the University of Chicago has the most co-citations. Notably, Gillies R.J. also ranks among the top 10 co-cited authors, underscoring his significant academic influence. As an exceptional interdisciplinary scientist often referred to as the “father of radiomics,” Gillies has made substantial contributions to clinical trials, image analytics, AI, and molecular & cellular studies. His pioneering work in radiomics has greatly enhanced clinical decision-making, particularly in lung cancer screening and diagnosis^[21,45]^.

Armato S.G., another prominent figure among the top 10 co-cited authors, has led landmark interdisciplinary and multicenter image-based projects. His research primarily focuses on computerized detection and evaluation of pulmonary nodules in thoracic CT scans, establishing him as a key contributor to the field^[46,47]^. This dual recognition of both Gillies and Armato not only reflects their outstanding academic output but also indicates their central positions in global scientific collaborative networks. These researchers often serve as intellectual hubs within the AI-medical imaging community, facilitating the standardization of methodologies and the diffusion of innovative ideas across institutions and borders. Their frequent involvement in international initiatives and guideline development processes further strengthens their leadership roles. The sustained academic impact of these scholars is also a product of the robust research environments in which they work. The United States, where both are based, has long maintained a supportive infrastructure for interdisciplinary research, with funding bodies such as the NIH and the National Cancer Institute actively promoting the convergence of medical imaging, artificial intelligence, and oncology. These agencies encourage the integration of domain-specific knowledge, fostering an environment where researchers from radiology, computer science, bioinformatics, and clinical medicine collaborate effectively. This convergence science approach is particularly essential in AI-based pulmonary nodule research, where translational success depends on the ability to bridge technical innovation and clinical relevance.

Despite the progress made, collaboration between countries remains limited. Economic disparities, inconsistent data governance policies, and variable research priorities continue to constrain meaningful international cooperation. High-income countries with advanced computational infrastructure and sustained public investment dominate the academic landscape, while low- and middle-income countries often face challenges in accessing the necessary technological, human, and financial resources. Bridging this gap will require not only enhanced global cooperation initiatives but also a shift toward equitable access to data platforms, algorithmic tools, and educational resources.

Lastly, the future of AI-based pulmonary nodule research hinges on cultivating a new generation of interdisciplinary scientists. These individuals must possess not only technical expertise in artificial intelligence but also a deep understanding of clinical contexts and imaging biology. Governments and academic institutions should prioritize interdisciplinary education and create incentive structures that reward collaboration across traditionally siloed domains. Such efforts will be key to accelerating innovation and ensuring that AI technologies effectively translate into improved diagnostic accuracy and patient care worldwide^[48]^.

### Visual analysis of journals and co-cited journals

Academic journals play a central role in the dissemination of scientific knowledge and scholarly communication. Analyzing the most influential journals in the field of AI-based pulmonary nodule research helps researchers identify high-impact sources, select appropriate outlets for publication, and stay abreast of frontier trends. This study found that *Radiology* stands out as the most influential journal, evidenced by its high co-citation frequency and IF. Meanwhile, *Academic Radiology* ranks highest in terms of betweenness centrality, suggesting its pivotal role as a hub that connects diverse research communities.

Further analysis revealed that 9 out of the top 10 co-cited journals (90%) belong to the Q1 category in the JCR, underscoring the dominant position of high-quality journals in shaping this research field. It is worth noting that most of these journals are published by leading academic publishers in Western countries, reflecting a “core–periphery” structure in the global academic publishing system. The dominance of these journals is closely linked to systematic advantages in scientific funding, peer-review mechanisms, editorial board composition, and the long-standing accumulation of academic reputation in developed countries.

From the perspective of knowledge dissemination, high-impact journals such as *Radiology* serve not only as platforms for publishing research findings, but also as intellectual hubs that facilitate interdisciplinary collaboration and innovation. By publishing high-level reviews, consensus guidelines, and multicenter research, these journals influence research direction-setting and even indirectly shape funding priorities. Their influence is supported by a comprehensive set of mechanisms, including rigorous editorial practices, strategic positioning, open access policies, and global collaboration networks.

Moreover, national research evaluation systems significantly influence journal selection. In many countries, career advancement and funding success are closely tied to publications in high-impact journals, which motivates scholars to prioritize submission to Q1 international journals. In China, for instance, the increasing emphasis on high-quality publications in national funding programs – such as the National Natural Science Foundation of China – has driven a steady rise in the number of Chinese scholars publishing in core international journals. In addition, the trends of open science and sustainable publishing are also reshaping the academic landscape. Many top journals have adopted open access models, enhanced the visibility and accessibility of research outputs while offered more equitable access to scientific knowledge for scholars in low- and middle-income countries^[48,49]^. This shift holds promise for fostering a more inclusive and collaborative global research environment.

In summary, influential journals in AI-based pulmonary nodule research not only serve as key vehicles for knowledge dissemination, but also exert profound influence over the structure and evolution of the scholarly communication ecosystem. Therefore, researchers should not only consider citation metrics but also be aware of the institutional, systemic, and global factors that shape academic influence, in order to strategically align their research output with the evolving dynamics of the field.

## Research hotspots and frontiers

### Hot spot 1: application of AI in pulmonary nodule detection

Keyword and reference analysis are powerful tools for identifying research hotspots and understanding the trajectory of academic fields. In our analysis using CiteSpace, we identified 17 keyword clusters, as shown in Figure 7. Combining the results of the literature analysis, our findings reveal a close connection between pulmonary nodule research and AI technologies, particularly machine learning (ML) and deep learning (DL). The most-cited reference was “End-to-end lung cancer screening with three-dimensional DL on low-dose chest computed tomography” by Tse D. *et al*, published in *Nature Medicine* (IF = 58.7) in 2019^[18]^, which focused on the potential of DL to reduce false-positive rates in pulmonary nodule detection and lung cancer screening. This phenomenon underscores the significant role of AI in enhancing diagnostic accuracy and efficiency.

#### Technical evolution of AI-based detection models

With the development of DL, particularly CNNs, the landscape of pulmonary nodule detection has undergone significant transformation. Early approaches based on handcrafted features and rule-based algorithms were prone to low generalizability and high false-positive rates. In contrast, CNN-based methods – such as 2D CNNs, 3D CNNs, and U-Net architectures – have shown superior performance in localizing nodules in LDCT scans, especially for small or irregularly shaped lesions^[50]^. Moreover, as research progresses, architectural strategies for detection have diversified. Broadly, two major paradigms have been explored in the context of AI-based object detection and increasingly adapted for medical imaging: (1) Two-stage detectors, such as Faster R-CNN, first generate candidate regions and then perform classification and refinement. These models offer high precision and are well-suited for complex nodule localization tasks, albeit with higher computational cost and longer inference time^[51]^; (2) One-stage detectors, such as YOLO (You Only Look Once) and SSD (Single Shot MultiBox Detector), perform detection and classification in a single pass, enabling real-time performance^[52,53]^. While initially developed for natural image detection, these models have been modified to process CT data, offering potential for fast triage systems in clinical workflows.

However, unlike general object detection, pulmonary nodules are three-dimensional entities often requiring spatial context. As a result, 3D CNNs and volumetric detection models have been increasingly adopted. These models can exploit the full 3D structure of nodules, improving both sensitivity and robustness. For instance, 3D U-Net variants have been used to segment and detect nodules across multiple slices with higher spatial consistency^[54]^.

More recently, attention-based architectures and transformer models have been introduced to better capture long-range spatial dependencies in volumetric data. These models dynamically weigh the importance of different regions within the CT volume, allowing more accurate detection of ambiguous or subtle nodules, especially in complex anatomical backgrounds^[55]^.

In summary, the technical evolution of AI-based pulmonary nodule detection has progressed from simple rule-based computer-aided detection (CADe) systems to complex DL architectures, with current research focusing on balancing accuracy, efficiency, and clinical adaptability. Future innovations are likely to involve hybrid approaches combining volumetric attention mechanisms, real-time inference, and integration with clinical metadata to improve generalizability and reduce false positives across diverse patient populations.

#### Current challenges to pulmonary nodule detection: data limitations and clinical relevance

Most current models are developed using public Western-centric datasets, which lack representation from underdeveloped regions or varied clinical environments. Furthermore, these datasets often exclude complex real-world cases such as overlapping nodules or CT images with artifacts. Building multicenter, multinational databases with standardized annotation protocols is essential for creating generalizable AI detection systems.

Although AI has demonstrated high sensitivity, specificity remains a major concern. Many systems produce excessive false positives, leading to unnecessary patient anxiety, follow-up imaging, and invasive procedures. For example, studies using LUNA16 reported false positive rates of up to 0.2 per scan^[56]^. To address this, recent models incorporate post-processing filters, contextual anatomical constraints, and ensemble learning to reduce false alarms^[57]^. Integrating clinical metadata (e.g. smoking history, age) with image-based models has also shown promise in prioritizing clinically relevant findings^[58]^. The practical adoption of AI detection tools must balance high sensitivity with low false positive rates and robust performance across diverse patient populations.

#### Future innovations: weak supervision, active learning, and transformers

A key barrier in detection model development is the scarcity of labeled data. Weakly supervised learning, where only partial or imprecise labels are used, has emerged as a promising solution^[59]^. Models trained with slice-level or scan-level labels can still learn to identify nodules without exhaustive pixel-wise annotation. Self-supervised learning and contrastive learning are also being explored to pre-train models using large unlabeled datasets^[60,61]^. Another underexplored area is active learning, where models iteratively select the most informative samples for annotation, maximizing efficiency and reducing labeling costs^[62]^. Combined with radiologist-in-the-loop systems, this could accelerate the creation of high-quality training datasets in resource-limited settings. Furthermore, transformer-based architectures, originally designed for natural language processing, are increasingly adapted for medical imaging^[63]^. These models capture long-range dependencies and have demonstrated competitive performance in recent studies on pulmonary nodule detection.

In summary, in comparison to traditional non-AI detection methods – such as manual interpretation by radiologists or classical CADe systems – AI-based approaches offer several advantages. First, AI systems can process and analyze large volumes of CT data rapidly and consistently, reducing inter-observer variability and human fatigue. Second, AI algorithms, especially DL models, are capable of learning hierarchical and abstract features directly from raw images, enabling the detection of subtle or atypical nodules that might be overlooked by human experts. Additionally, AI models can integrate multimodal data (e.g. imaging, clinical history), thereby enhancing risk stratification and decision support. However, AI also presents some disadvantages when compared to traditional methods. Unlike experienced radiologists, AI systems often lack transparent reasoning processes, leading to challenges in interpretability and clinical trust. Furthermore, AI performance is highly dependent on the quality and representativeness of training data. In cases with image artifacts, rare anatomical variants, or insufficient training on diverse populations, AI systems may underperform or generate misleading results. Thus, while AI offers transformative potential, it is not yet a replacement for expert clinical judgment, but rather a tool that complements and augments it.

Besides, AI-based pulmonary nodule detection has evolved rapidly, with DL significantly outperforming traditional methods. However, limitations in data diversity, false positive control, and interpretability remain key challenges. Future research should prioritize the development of multi-institutional datasets, hybrid learning strategies (e.g. active + weak supervision), and novel model architectures (e.g. Vision Transformers) to build robust and clinically useful detection tools. These advancements will be crucial for the widespread and equitable adoption of AI-assisted lung cancer screening across varied healthcare environments.

### Hot spot 2: application of AI in pulmonary nodule classification

Following detection, the classification of pulmonary nodules into benign or malignant plays a critical role in clinical decision-making, influencing the need for surgical intervention, biopsy, or follow-up. Therefore, analyzing the application of AI in pulmonary nodule classification undoubtedly is important for researchers engaged in this field.

#### Approaches to pulmonary nodule classification

Current classification approaches can be broadly divided into two categories: traditional ML methods based on radiomic feature extraction, and DL methods that automatically learn discriminative patterns from raw imaging data. Radiomics-based ML models typically extract predefined features – such as texture, shape, margin, and intensity – from segmented nodules and use classifiers like support vector machines (SVM), random forests (RF), or logistic regression to distinguish benign from malignant lesions^[64]^. These models offer interpretability and are effective in small datasets, but they often suffer from feature selection bias and lack adaptability to complex patterns. DL models, particularly CNNs, have demonstrated superior performance by learning hierarchical features directly from CT images^[65]^. Architectures such as ResNet, DenseNet, and EfficientNet are commonly employed. Some studies also explore dual-path CNNs that combine local nodule features and global lung context to improve classification accuracy. However, these models require large annotated datasets and are often regarded as “black boxes,” limiting their clinical transparency and trustworthiness.

#### Challenges in real-world deployment

Despite technical advancements, several key challenges hinder the clinical translation of AI-based classification systems: (1) Class imbalance: Malignant nodules are far less prevalent than benign ones, which can lead to biased models unless carefully addressed through resampling or cost-sensitive learning strategies. (2) Weak or noisy labels: Many labels are based on radiological suspicion or short-term follow-up rather than pathological confirmation, introducing uncertainty in training data. (3) Domain shift: Models trained on specific institutions or image acquisition protocols may not generalize well to external datasets, underscoring the need for multicenter validation and domain adaptation techniques. (4) Lack of interpretability: Most DL models do not provide explanations for their predictions, which poses a barrier to clinician acceptance in high-stakes diagnostic contexts.

#### Emerging innovations and future directions

Several promising research directions aim to address the aforementioned limitations and enhance the robustness and clinical utility of classification models: (1) Explainable AI (XAI): Techniques such as Grad-CAM, LIME, and SHAP are being integrated to highlight influential image regions and provide clinicians with visual justifications for model decisions. However, standardized interpretability protocols are still lacking^[66]^. (2) Few-shot and self-supervised learning: These approaches enable models to generalize from limited labeled samples, which is particularly valuable for rare malignancies or underrepresented populations^[67]^. (3) Federated learning: This decentralized training method allows institutions to collaboratively develop classification models without sharing raw data, preserving patient privacy and overcoming data fragmentation across centers^[68]^. (4) Cross-modality fusion: Combining CT imaging with other modalities such as PET, MRI, or radiology reports may further enhance classification accuracy and reduce diagnostic uncertainty.

Besides, recent studies are increasingly moving toward multimodal AI frameworks that combine imaging data with patient-specific clinical parameters (e.g. age, smoking history, family history) and even genetic or molecular profiles. Such integrated models have demonstrated improved diagnostic performance and represent a step toward personalized, precision medicine.

In summary, AI-based classification of pulmonary nodules has shown substantial progress, evolving from radiomics-driven ML models to deep and multimodal architectures. While performance metrics in controlled datasets are promising, challenges such as class imbalance, interpretability, and domain generalization remain. Future research should focus on developing explainable, data-efficient, and clinically validated models that can be integrated seamlessly into diagnostic workflows. These efforts will be pivotal in enabling AI to assist clinicians in making more accurate, transparent, and personalized decisions for pulmonary nodule management.

### Hot spot 3: AI in malignancy risk prediction and growth modeling

Moreover, in our analysis of keyword clustering (Fig. 7), we also found terms related to AI algorithms for assessing pulmonary nodule malignancy risk, such as the DCNN model, delta feature, and cancer diagnosis. Thus, it is essential to conduct a systematic discussion on AI-driven malignancy risk prediction and growth modeling for pulmonary nodules in the following sections.

#### Malignancy risk prediction based on static imaging

Traditional risk models for pulmonary nodules, such as the Mayo Clinic and Brock models, incorporate clinical features (e.g. age, smoking status, nodule size) to estimate malignancy probability. With the advent of AI, more sophisticated approaches have emerged. Radiomics-based models extract quantitative features (e.g. texture, shape, edge sharpness) from CT images and use ML algorithms such as RF and SVM to predict malignancy. These models have demonstrated improved performance over human interpretation in several studies^[69]^.

More recently, DL approaches have been employed to learn high-dimensional features directly from raw images without explicit feature engineering. CNN-based models can infer malignancy probability end-to-end, offering scalability and automation. For instance, Google’s DL model published by Tse *et al* in *Nature Medicine* employed a 3D CNN to predict lung cancer from low-dose CT scans with performance on par with expert radiologists^[18]^. However, these models remain data-hungry and are limited by their black-box nature and generalization gaps across populations.

#### Growth modeling using longitudinal imaging

Pulmonary nodules are inherently dynamic. Nodules that increase in size, change in density, or exhibit morphological evolution over time are more likely to be malignant. Growth modeling aims to quantify such temporal changes using serial CT scans. A commonly used metric is volume doubling time (VDT), which correlates with tumor aggressiveness. However, manual or rule-based VDT assessment is prone to inconsistency.

AI offers novel solutions for longitudinal analysis. Some models use delta-radiomics, which captures changes in radiomic features between two time points to estimate progression. Others adopt recurrent neural networks, long short-term memory networks, or even emerging transformer-based temporal models to model sequences of CT scans^[70,71]^. These time-aware models can learn nodule evolution patterns and forecast future malignancy risk or progression trajectories.

Recent studies have also begun to integrate clinical metadata (e.g. demographics, lab values) and even genomic information with temporal imaging features to create multimodal predictive frameworks. These integrated systems hold promise for personalized surveillance strategies tailored to individual patients’ risk profiles^[72]^.

#### Challenges and future perspectives

Despite their promise, predictive and growth modeling tasks face several barriers. First, longitudinal imaging data is difficult to obtain, and are often inconsistent in timing and imaging parameters. Second, label noise and outcome uncertainty (e.g. no biopsy confirmation) reduce model reliability. Third, temporal models are complex and require substantial computational resources and interpretability mechanisms.

Going forward, promising directions include: (1) Federated temporal learning across institutions to expand training datasets without compromising privacy. (2) Few-shot prediction models trained on limited follow-up data. (3) Time-aware XAI, which helps clinicians understand how nodule evolution influences malignancy predictions.

In summary, AI-driven malignancy risk prediction and growth modeling represent powerful tools for optimizing pulmonary nodule management. Static imaging models estimate malignancy probability at a given time point, while longitudinal approaches assess progression trends, offering a temporal dimension to diagnosis. Together, they form a continuum of AI support, from detection to dynamic risk stratification. Overcoming data fragmentation and improving model transparency will be crucial for their successful integration into routine clinical workflows.

### Hot spot 4: AI-driven development of drug efficacy evaluation metrics may be a future direction for pulmonary nodule treatment research

Although surgery is the main intervention method for high-risk pulmonary nodules, not all patients are able to tolerate or are willing to undergo surgical treatment. For these patients, medication is necessary^[58]^. Therefore, while ensuring safety, it is essential to develop drugs that could control, reduce, or eliminate pulmonary nodules and lower their risk of malignancy. Scientific and objective efficacy evaluation metrics are the foundation of drug development. However, there is currently no universally recognized method for evaluating the efficacy of pulmonary nodule treatments, which limits the development of research on the treatment of pulmonary nodules.

AI has the potential to address these challenges, but it relies on advancements in algorithms. These algorithms must enhance the understanding of pulmonary nodule imaging and incorporate more nuanced features, to improve their diagnostic accuracy and predictive capability. With AI support, the accuracy of pulmonary nodule diagnosis has improved significantly compared to traditional assessment methods. If we can leverage AI to develop methods for evaluating the therapeutic efficacy of pulmonary nodules and integrating these methods with drug discovery, we can potentially provide a nonsurgical treatment alternative and prevent the malignant transformation of high-risk nodules. This would be of great significance for early-stage lung cancer research. In fact, existing evidence suggests that early high-risk pure ground-glass nodules could achieve remission through pharmacological treatment^[73]^. Genome mapping studies have also revealed the molecular mechanisms of pulmonary nodule pathogenesis and identified potential drug targets^[74]^. Additionally, existing evidence demonstrates that radiomics, an AI-driven technology, can assess tumor treatment efficacy more effectively than conventional evaluation methods. A study published in *Radiology* revealed that radiomics enables accurate and robust stratification of survival risks in stage IA pure-solid non-small cell lung cancer (NSCLC) patients. Moreover, a study on locally advanced rectal cancer demonstrated that radiomics outperformed experienced radiologists in classifying pathological complete response following neoadjuvant chemo-radiotherapy^[75]^. This capability makes efficacy evaluation metrics offering promise to facilitate personalized management strategies for early-stage lung cancer patients^[75]^.

Despite the compelling evidence highlighting the immense potential of AI in evaluating treatment efficacy for pulmonary nodules, relevant evidence remains limited, and no comprehensive expert consensus has been established on efficacy evaluation. Further research is needed in this field. Minimizing human bias and variability is a significant advantage, particularly in clinical settings where accurate and timely decision-making is crucial. Given current research trends, if we were able to leverage AI algorithms to develop tailored efficacy evaluation methods for pulmonary nodules, we may achieve more objective, accurate, and comprehensive assessments. This would undoubtedly play a crucial role in advancing pharmacological treatment research for pulmonary nodules. However, we still need high-level evidence from clinical studies, as well as further studies to elucidate the underlying mechanisms. This research direction is highly relevant and impactful.

Moreover, in terms of prognosis, AI also demonstrates significant advantages. Radiomics features associated with the tumor immune micro-environment extracted by Mazzaschi *et al*^[76]^, when combined with clinical pathological characteristics, could effectively predict the prognosis of NSCLC patients after surgical resection. Wang *et al*^[77]^ have used PyRadiomics to extract radiomic features from the two-dimensional, three-dimensional, and peritumoral regions of tumors, selecting the best features to perform personalized survival risk stratification for NSCLC patients with clinical and pathological stage IA. The AUCs for 3-year and 5-year survival in the external validation cohort were 0.76 and 0.75, respectively. The graph neural network, a type of neural network, captures dependencies within a graph by transferring information between nodes, making it a connectionist model^[78]^. Lian *et al*^[79]^ have proposed a model based on graph neural networks, which utilizes a visual transformer to extract CT image features and embeds both imaging and non-imaging data into the neural network. This model classifies the overall survival and recurrence risk of early-stage NSCLC patients, and its performance on external datasets significantly outperforms TNM staging. With this research foundation, it becomes possible to use AI technology to evaluate the efficacy of drug treatments for pulmonary nodules in the future.

Particularly in under-resourced regions, where radiological expertise is limited, AI-driven diagnostic and efficacy assessment tools can provide scalable, cost-effective alternatives to support early lung cancer detection and treatment evaluation. This reflects the growing importance of interdisciplinary solutions in global health equity and underscores the value of applying AI technologies to address real-world clinical needs.

### Summary

AI has profoundly reshaped key tasks in pulmonary nodule management, including detection, classification, malignancy risk prediction, and therapeutic response evaluation. As previously discussed, a variety of AI techniques have been applied across these domains. Broadly, they can be categorized into three major types: First, traditional ML models, such as SVM and RF, rely on handcrafted radiomic features. These models offer interpretability and perform reasonably well with small-to-moderate datasets but are often limited in adapting to complex image patterns. Second, DL models, especially CNNs, have become the mainstream approach. These models extract high-dimensional features directly from raw images in an end-to-end fashion, leading to superior accuracy in detection and classification tasks. However, their “black-box” nature, high data requirements, and limited interpretability pose challenges for clinical translation. Third, emerging foundation models, including vision transformers, graph neural networks, and multimodal fusion frameworks, are gaining attention. These models can capture long-range dependencies and spatiotemporal dynamics while integrating diverse data modalities such as imaging, clinical variables, and genomic information. Despite their promise, these approaches remain in the exploratory phase and require large-scale validation and robust evaluation frameworks before widespread adoption.

Besides, these AI paradigms differ substantially in terms of technical complexity, interpretability, and clinical usability. The choice of methodology should be tailored to the specific clinical task, available data resources, and deployment environment. Compared with conventional rule-based or radiologist-dependent diagnostic systems, AI-based methods offer notable advantages: improved diagnostic efficiency, reduced human error, and enhanced individualized risk assessment. Nonetheless, the clinical implementation of AI remains contingent on addressing challenges such as multi-institutional data heterogeneity, lack of model transparency, limitations in generalizability, and the need for equitable and privacy-preserving frameworks.

Looking ahead, the clinical translation of AI in pulmonary nodule management will depend on three key strategies: (1) developing hybrid models that combine high performance with interpretability through visual outputs; (2) constructing multi-modal, multi-center, and continuously updated data ecosystems to enhance model generality; and (3) exploring AI-guided personalized treatment planning and response assessment for high-risk patient’s ineligible for surgery.

Overall, establishing a structured and systematic understanding of AI methodologies will help clinicians, researchers, and policymakers better assess the current limitations and future potential of AI. This, in turn, will facilitate the shift from algorithmic innovation to meaningful clinical integration in pulmonary nodule management. In terms of practical applications, the results of this study provide actionable insights for multiple stakeholders. For clinicians, identifying AI research trends – particularly in diagnostic accuracy and malignancy risk prediction – can support informed decision-making in early lung cancer screening and follow-up strategies. For AI developers and researchers, the highlighted technical limitations such as lack of annotated datasets, interpretability issues, and data heterogeneity offer clear directions for future algorithm refinement. Furthermore, for healthcare administrators and policymakers, recognizing the global research distribution helps inform investment strategies, promote international collaboration, and guide the development of regulatory and ethical frameworks for clinical AI implementation. These findings, therefore, extend beyond academic value and serve as a roadmap for bridging the gap between cutting-edge research and real-world medical applications.

## Limitations

Admittedly, there are still some limitations. First, this study only selected articles and reviews in the WoSCC database, but due to the limitations of bibliometric software, it was difficult to merge various databases for analysis; we presented reasons for choosing WoSCC as our database in the methods section. Secondly, our study might overlook some important non-English publications, leading to research bias and decreased inclusivity. Finally, since the continuous update of the database, recent high-quality papers might be underestimated for their undesirable number of citations.

## Conclusion

To our knowledge, this is the first comprehensive analysis of study related to AI in pulmonary nodules from 2005 to 2024 by bibliometrics. The number of publications on AI-based pulmonary nodules research has been growing in the past 20 years, especially after 2014. China and the United States have contributed the most to this field. Shanghai Jiao Tong University was the most productive institution. R.J. Gillies (Moffitt Cancer Center) and S.G. Armato III (University of Chicago) were identified as the most influential authors. In conclusion, the prominent research areas that overlap in AI and pulmonary nodule research concentrate on the subsequent topics: (1) Application of AI in pulmonary nodule detection and classification; (2) AI in malignancy risk prediction and growth modeling; (3) AI-driven development of drug efficacy evaluation metrics may be a future direction for pulmonary nodule treatment research. This timely review scrutinizes research trends and hotspots related to AI and pulmonary nodules, which could progress the field and form the basis for forthcoming studies.

## Data Availability

The raw data supporting the conclusions of this article will be made available by the authors, without undue reservation.

## References

[1] Sung H Ferlay J Siegel RL. Global cancer statistics 2020: GLOBOCAN estimates of incidence and mortality worldwide for 36 cancers in 185 Countries. CA Cancer J Clin 2021;71:209–49. 33538338 10.3322/caac.21660

[2] Kay FU Kandathil A Batra K Saboo SS Abbara S Rajiah P. Revisions to the tumor, node, metastasis staging of lung cancer (8(th) edition): rationale, radiologic findings and clinical implications. World J Radiol 2017;9:269–79. 28717413 10.4329/wjr.v9.i6.269 PMC5491654

[3] Kinsinger LS Anderson C Kim J. Implementation of lung cancer screening in the veterans health administration. JAMA Intern Med 2017;177:399–406. 28135352 10.1001/jamainternmed.2016.9022

[4] National Lung Screening Trial Research T. Aberle DR Adams AM Berg CD. Reduced lung-cancer mortality with low-dose computed tomographic screening. N Engl J Med 2011;365:395–409. 21714641 10.1056/NEJMoa1102873 PMC4356534

[5] Adams SJ Stone E Baldwin DR Vliegenthart R Lee P Fintelmann FJ. Lung cancer screening. Lancet 2023;401:390–408. 36563698 10.1016/S0140-6736(22)01694-4

[6] Wood DE Kazerooni EA Aberle D. NCCN guidelines® insights: lung cancer screening, version 1.2022: featured updates to the NCCN guidelines. J National Compr Cancer Network 2022;20:754–64. 10.6004/jnccn.2022.0036 35830884

[7] Gong J Liu J Hao W. A deep residual learning network for predicting lung adenocarcinoma manifesting as ground-glass nodule on CT images. Eur Radiol 2020;30:1847–55 31811427 10.1007/s00330-019-06533-w

[8] Lin S Chen Y Yang L Zhou J. Pain, fatigue, disturbed sleep and distress comprised a symptom cluster that related to quality of life and functional status of lung cancer surgery patients. J Clin Nurs 2013;22:1281–90 23574291 10.1111/jocn.12228

[9] Fagundes CP Shi Q Vaporciyan AA. Symptom recovery after thoracic surgery: measuring patient-reported outcomes with the MD Anderson Symptom Inventory. J Thoracic Cardiovasc Surg 2015;150:613–19. 10.1016/j.jtcvs.2015.05.057 PMC4554973 26088408

[10] Licker M De Perrot M Hühn L. Perioperative mortality and major cardio-pulmonary complications after lung surgery for non-small cell carcinoma. Eur J Cardiothorac Surg 1999;15:314–19. 10333029 10.1016/s1010-7940(99)00006-8

[11] Im Y Park HY Shin S. Prevalence of and risk factors for pulmonary complications after curative resection in otherwise healthy elderly patients with early stage lung cancer. Respir Res 2019;20:1–9 31272446 10.1186/s12931-019-1087-x PMC6610954

[12] Rajapakse P. An update on survivorship issues in lung cancer patients. World J Oncol 2021;12:45. 34046098 10.14740/wjon1368 PMC8139739

[13] Kim RY Oke JL Dotson TL Bellinger CR Vachani A. Effect of an artificial intelligence tool on management decisions for indeterminate pulmonary nodules. Respirology 2023;28:582–84. 37017091 10.1111/resp.14502 PMC12910410

[14] Binczyk F Prazuch W Bozek P Polanska J. Radiomics and artificial intelligence in lung cancer screening. Transl Lung Cancer Res 2021;10:1186 33718055 10.21037/tlcr-20-708 PMC7947422

[15] Park S Lee SM Kim N. Application of deep learning–based computer-aided detection system: detecting pneumothorax on chest radiograph after biopsy. Eur Radiol 2019;29:5341–48. 30915557 10.1007/s00330-019-06130-x

[16] Li X Hu B Li H You B. Application of artificial intelligence in the diagnosis of multiple primary lung cancer. Thoracic Cancer 2019;10:2168–74 31529684 10.1111/1759-7714.13185 PMC6825907

[17] Ding H Xia W Zhang L. CT-based deep learning model for invasiveness classification and micropapillary pattern prediction within lung adenocarcinoma. Front Oncol 2020;10:1186. 32775302 10.3389/fonc.2020.01186 PMC7388896

[18] Ardila D Kiraly AP Bharadwaj S. End-to-end lung cancer screening with three-dimensional deep learning on low-dose chest computed tomography. Nat Med 2019;25:954–61 31110349 10.1038/s41591-019-0447-x

[19] Rajpurkar P Irvin J Ball RL. Deep learning for chest radiograph diagnosis: a retrospective comparison of the CheXNeXt algorithm to practicing radiologists. PLoS Med 2018;15:e1002686. 30457988 10.1371/journal.pmed.1002686 PMC6245676

[20] Nasrullah N Sang J Alam MS Mateen M Cai B Hu H. Automated lung nodule detection and classification using deep learning combined with multiple strategies. Sensors 2019;19:3722. 31466261 10.3390/s19173722 PMC6749467

[21] Gillies RJ Schabath MB. Radiomics improves cancer screening and early detection. Cancer Epidemiol Biomarkers Prev 2020;29:2556–67. 32917666 10.1158/1055-9965.EPI-20-0075 PMC12306495

[22] Pinsky PF Gierada DS Black W. Performance of lung-RADS in the national lung screening trial: a retrospective assessment. Ann Internal Med 2015;162:485–91. 25664444 10.7326/M14-2086 PMC4705835

[23] Setio AA Traverso A De Bel T. Validation, comparison, and combination of algorithms for automatic detection of pulmonary nodules in computed tomography images: the LUNA16 challenge. Med Image Anal 2017;42:1–3. 28732268 10.1016/j.media.2017.06.015

[24] López Alcolea J Fernández Alfonso A Cano Alonso R. Diagnostic performance of Artificial Intelligence in chest radiographs referred from the emergency department. Diagnostics 2024;14:2592. 39594258 10.3390/diagnostics14222592 PMC11592727

[25] MacMahon H Naidich DP Goo JM. Guidelines for management of incidental pulmonary nodules detected on CT images: from the fleischner society 2017. Radiology 2017;284:228–43 28240562 10.1148/radiol.2017161659

[26] Yun T Cosentino J Behsaz B. Unsupervised representation learning on high-dimensional clinical data improves genomic discovery and prediction. Nature Genet 2024;56:1604–13 38977853 10.1038/s41588-024-01831-6 PMC11319202

[27] Rudin C. Stop explaining black box machine learning models for high stakes decisions and use interpretable models instead. Nature Mach Intell 2019;1:206–15 35603010 10.1038/s42256-019-0048-x PMC9122117

[28] Agha RA Mathew G Rashid R. Transparency in the reporting of artificial intelligence–the TITAN guideline. Prem J Sci 2025;10:100082.

[29] Burstein HJ Curigliano G Thürlimann B. Customizing local and systemic therapies for women with early breast cancer: the St. Gallen international consensus guidelines for treatment of early breast cancer 2021. Ann Oncol 2021;32:1216–35. 34242744 10.1016/j.annonc.2021.06.023 PMC9906308

[30] McDonald ES Clark AS Tchou J Zhang P Freedman GM. Clinical diagnosis and management of breast cancer. J Nucl Med 2016;57:9S–16S. 26834110 10.2967/jnumed.115.157834

[31] Chen C. Science mapping: a systematic review of the literature. J Data Inf Sci 2017;2:1–40

[32] Wang C Zhang Y Zhang Y Li B. A bibliometric analysis of gastric cancer liver metastases: advances in mechanisms of occurrence and treatment options. Int J Surg 2024;110:2288–99. 38215249 10.1097/JS9.0000000000001068 PMC11020032

[33] Li H Han Z Wu H. Artificial intelligence in surgery: evolution, trends, and future directions. Int J Surg 2025;111:2101–11. 39693484 10.1097/JS9.0000000000002159

[34] Kim S Yeganova L Wilbur WJ. Meshable: searching PubMed abstracts by utilizing MeSH and MeSH-derived topical terms. Bioinformatics 2016;32:3044–46. 27288493 10.1093/bioinformatics/btw331 PMC5039918

[35] Jelercic S Lingard H Spiegel W Pichlhöfer O Maier M. Assessment of publication output in the field of general practice and family medicine and by general practitioners and general practice institutions. Family Practice 2010;27:582–89. 20554654 10.1093/fampra/cmq032

[36] Page MJ McKenzie JE Bossuyt PM. The PRISMA 2020 statement: an updated guideline for reporting systematic reviews. Bmj 2021;372:n71. 10.1136/bmj.n71 PMC8005924 33782057

[37] Zhou Z Siddiquee MM Tajbakhsh N Liang J. Unet++: redesigning skip connections to exploit multiscale features in image segmentation. IEEE Transact Med Imaging 2019;39:1856–67. 10.1109/TMI.2019.2959609 PMC7357299 31841402

[38] Sun Y Li C Jin L. Radiomics for lung adenocarcinoma manifesting as pure ground-glass nodules: invasive prediction. Eur Radiol 2020;30:3650–59 32162003 10.1007/s00330-020-06776-y PMC7305264

[39] National Lung Screening Trial Research Team. Reduced lung-cancer mortality with low-dose computed tomographic screening. N Engl J Med 2011;365:395–409. 21714641 10.1056/NEJMoa1102873 PMC4356534

[40] Li N Wang L Hu Y. Global evolution of research on pulmonary nodules: a bibliometric analysis. Future Oncol 2021;17:2631–45. 33880950 10.2217/fon-2020-0987

[41] Center for Security and Emerging Technology. Internet + Artificial Intelligence three-year action and implementation plan [Internet]. Georgetown University. https://cset.georgetown.edu/publication/internet-artificial-intelligence-three-year-action-and-implementation-plan/

[42] AI Newsletter. AIx newsletter – may 1 issue [Internet]. https://www.ainewsletter.com/newsletters/aix_0501/#w

[43] Batra U Nathany S Nath SK. AI-based pipeline for early screening of lung cancer: integrating radiology, clinical, and genomics data. Lancet Reg Health-Southeast Asia 2024;24:100352. 38756151 10.1016/j.lansea.2024.100352 PMC11096686

[44] Mazzone PJ Silvestri GA Souter LH. Screening for lung cancer: CHEST guideline and expert panel report. Chest 2021;160:e427–94. 34270968 10.1016/j.chest.2021.06.063 PMC8727886

[45] Hawkins S Wang H Liu Y. Predicting malignant nodules from screening CT scans. J Thorac Oncol 2016;11:2120–28. 27422797 10.1016/j.jtho.2016.07.002 PMC5545995

[46] Armato SG III McLennan G Bidaut L. The lung image database consortium (LIDC) and image database resource initiative (IDRI): a completed reference database of lung nodules on CT scans. Med Phys 2011;38:915–31 21452728 10.1118/1.3528204 PMC3041807

[47] Armato SG Giger ML Moran CJ Blackburn JT Doi K MacMahon H. Computerized detection of pulmonary nodules on CT scans. Radiographics 1999;19:1303–11 10489181 10.1148/radiographics.19.5.g99se181303

[48] Xu J Xue K Zhang K. Current status and future trends of clinical diagnoses via image-based deep learning. Theranostics 2019;9:7556. 31695786 10.7150/thno.38065 PMC6831476

[49] Greussing E Kuballa S Taddicken M Schulze M Mielke C Haux R. Drivers and obstacles of open access publishing. A qualitative investigation of individual and institutional factors. Front Commun 2020;5:587465.

[50] Yang S Lim SH Hong JH Park JS Kim J Kim HW. Deep learning-based lung cancer risk assessment using chest computed tomography images without pulmonary nodules ≥ 8 mm. Transl Lung Cancer Res 2025;14:150–62. 39958220 10.21037/tlcr-24-882 PMC11826273

[51] Zamanidoost Y Ould-Bachir T, and Martel S. OMS-CNN: optimized multi-scale CNN for lung nodule detection based on faster R-CNN. IEEE J Biomed Health Inform 2024;29:2148–60. 10.1109/JBHI.2024.3507360 40030300

[52] Santone A Mercaldo F Brunese L. A method for real-time lung nodule instance segmentation using deep learning. Life 2024;14:1192. 39337974 10.3390/life14091192 PMC11433569

[53] Hanaoka S Nomura Y Yoshikawa T. Detection of pulmonary nodules in chest radiographs: novel cost function for effective network training with purely synthesized datasets. Int J Comput Assisted Radiol Surg 2024;19:1991–2000 10.1007/s11548-024-03227-7 PMC11442563 39003437

[54] Kashyap M Wang X Panjwani N. Automated deep learning-based detection and segmentation of lung tumors at CT. Radiology 2025;314:e233029. 39835976 10.1148/radiol.233029 PMC11783160

[55] Dedeken S Conze PH Pieters VD. Trustworthy AI for stage IV non-small cell lung cancer: automatic segmentation and uncertainty quantification. Computerized Med Imaging Graphics 2025;13:102567 10.1016/j.compmedimag.2025.102567 40381569

[56] Gu Y Chi J Liu J. A survey of computer-aided diagnosis of lung nodules from CT scans using deep learning. Comput Biol Med 2021;137:104806. 34461501 10.1016/j.compbiomed.2021.104806

[57] Li Y Hui L Wang X Zou L Chua S. Lung nodule detection using a multi-scale convolutional neural network and global channel spatial attention mechanisms. Sci Rep 2025;15:12313. 40210738 10.1038/s41598-025-97187-w PMC11986029

[58] Lin CY Guo SM Lien JJ. Combined model integrating deep learning, radiomics, and clinical data to classify lung nodules at chest CT. La Radiol Med 2024;129:56–69 10.1007/s11547-023-01730-6 PMC10808169 37971691

[59] Djahnine A Jupin-Delevaux E Nempont O. Weakly-supervised learning-based pathology detection and localization in 3D chest CT scans. Med Phys 2024;51:8272–82 39140793 10.1002/mp.17302

[60] Yu K Sun L Chen J Reynolds M Chaudhary T Batmanghelich K. DrasCLR: a self-supervised framework of learning disease-related and anatomy-specific representation for 3D lung CT images. Med Image Anal 2024;92:103062. 38086236 10.1016/j.media.2023.103062 PMC10872608

[61] He F Liu K Yang Z. Detection of genes and gene relations from biological pathway figures through image-text contrastive learning. IEEE J Biomed Health Inform 2024;28:5007–19. pathclip. 38568768 10.1109/JBHI.2024.3383610 PMC11363067

[62] Wu X Chen C Zhong M Wang J Shi J. COVID-AL: the diagnosis of COVID-19 with deep active learning. Med Image Anal 2021;68:101913. 33285482 10.1016/j.media.2020.101913 PMC7689310

[63] Amoros M Curado M Vicent JF. Evaluating super-resolution models in biomedical imaging: applications and performance in segmentation and classification. J Imaging 2025;11:104. 40278020 10.3390/jimaging11040104 PMC12027580

[64] Sun T Wang J Li X. Comparative evaluation of support vector machines for computer aided diagnosis of lung cancer in CT based on a multi-dimensional data set. Comput Methods Programs Biomed 2013;111:519–24. 23727300 10.1016/j.cmpb.2013.04.016

[65] Oncu E Ciftci F. Multimodal AI framework for lung cancer diagnosis: integrating CNN and ANN models for imaging and clinical data analysis. Comput Biol Med 2025;193:110488. 40449048 10.1016/j.compbiomed.2025.110488

[66] Ullah N Guzmán-Aroca F Martínez-Álvarez F De Falco I Sannino G. A novel explainable AI framework for medical image classification integrating statistical, visual, and rule-based methods. Med Image Anal 2025;6:103665 10.1016/j.media.2025.103665 40505210

[67] Liu J Qi L Xu Q. A self-supervised learning-based fine-grained classification model for distinguishing malignant from benign subcentimeter solid pulmonary nodules. Acad Radiol 2024;31:4687–95. 38777719 10.1016/j.acra.2024.05.002

[68] Shaikh AA Bhargavi MS Kumar C P. Weighted aggregation through probability based ranking: an optimized federated learning architecture to classify respiratory diseases. Comput Methods Programs Biomed 2023;242:107821. 37776709 10.1016/j.cmpb.2023.107821

[69] Gao N Tian S Li X. Three-dimensional texture feature analysis of pulmonary nodules in CT images: lung cancer predictive models based on support vector machine classifier. J Digi Imaging 2020;33:414–22 10.1007/s10278-019-00238-8 PMC7165221 31529236

[70] Xu Y Hosny A Zeleznik R. Deep learning predicts lung cancer treatment response from serial medical imaging. Clin Cancer Res 2019;25:3266–75. 31010833 10.1158/1078-0432.CCR-18-2495 PMC6548658

[71] Liu X Wang M Aftab R. Study on the prediction method of long-term benign and malignant pulmonary lesions based on lstm. Front Bioeng Biotechnol 2022;10:791424. 35309999 10.3389/fbioe.2022.791424 PMC8924408

[72] Wang L Zhang M Pan X. Integrative serum metabolic fingerprints based multi-modal platforms for lung adenocarcinoma early detection and pulmonary nodule classification. Adv Sci 2022;9:2203786 10.1002/advs.202203786 PMC9731719 36257825

[73] Postmus PE Kerr KM Oudkerk M. ESMO guidelines committee. Early and locally advanced non-small-cell lung cancer (NSCLC): ESMO clinical practice guidelines for diagnosis, treatment and follow-up. Ann Oncol 2017;28:iv1–21 28881918 10.1093/annonc/mdx222

[74] Liang P Peng M Tao J. Development of a genome atlas for discriminating benign, preinvasive, and invasive lung nodules. MedComm 2024;5:e644 39036344 10.1002/mco2.644 PMC11258453

[75] Cheng B Li C Li J. The activity and immune dynamics of PD-1 inhibition on high-risk pulmonary ground glass opacity lesions: insights from a single-arm, phase II trial. Signal Transduct Target Ther 2024;9:93. 38637495 10.1038/s41392-024-01799-z PMC11026465

[76] Mazzaschi G Milanese G Pagano P. Integrated CT imaging and tissue immune features disclose a radio-immune signature with high prognostic impact on surgically resected NSCLC. Lung Cancer 2020;144:30–39. 32361033 10.1016/j.lungcan.2020.04.006

[77] Wang T She Y Yang Y. Radiomics for survival risk stratification of clinical and pathologic stage IA pure-solid non–small cell lung cancer. Radiology 2022;302:425–34 34726531 10.1148/radiol.2021210109

[78] Bianchi FM Grattarola D Livi L Alippi C. Graph neural networks with convolutional arma filters. IEEE Trans Pattern Anal Mach Intell 2021;44:3496–507. 10.1109/TPAMI.2021.3054830 33497331

[79] Lian J Deng J Hui ES. Early stage NSCLS patients’ prognostic prediction with multi-information using transformer and graph neural network model. Elife 2022;11:e80547. 36194194 10.7554/eLife.80547 PMC9531948

